# Integrated cytomembrane proteomics identifies EpCAM/MGST1 as therapeutic targets in metastatic laryngeal carcinoma

**DOI:** 10.3389/fgene.2025.1615570

**Published:** 2025-07-24

**Authors:** Suling Zhuang, Xiaobo Wu, Xiaohuang Lin, Zhihan Li, Di Gan, Shixin Wang, Xue Lin, Gongbiao Lin, Miao Gao

**Affiliations:** ^1^Department of Otorhinolaryngology Head and Neck Surgery, Fujian Institute of Otorhinolaryngology, The First Affiliated Hospital, Fujian Medical University, Fuzhou, China; ^2^Department of Otorhinolaryngology Head and Neck Surgery, National Regional Medical Center, Binhai Campus of the First Affiliated Hospital, Fujian Medical University, Fuzhou, China; ^3^Fujian Provincial Clinical Medical Research Center for Ear, Nose and Throat Difficulty Diseases, The First Affiliated Hospital, Fujian Medical University, Fuzhou, China; ^4^Key Laboratory of Ministry of Education for Gastrointestinal Cancer, School of Basic Medical Sciences, Fujian Medical University, Fuzhou, China; ^5^Department of Gastrointestinal Surgery, Gutian County Hospital of Fujian Province, Ningde, Fujian, China

**Keywords:** lymph node metastasis, LSCC, cytomembrane protein, EpCAM, MGST1 lymph node metastasis, MGST1

## Abstract

**Background:**

Lymph node metastasis plays a crucial role in cancer recurrence and survival, however, the underlying molecular mechanism and biomarkers in laryngeal carcinoma remain poorly characterized. While cytomembrane proteins represent attractive therapeutic targets due to their accessibility, the identification of tractable candidates for precision therapy remains challenging.

**Methods:**

This study aimed to identify potential therapeutic targets for laryngeal squamous cell carcinoma (LSCC) with lymph node metastasis through cytomembrane proteome profiling. We conducted a comprehensive multi-omics analysis in 158 LSCC cases from TCGA (111 patients) and CPTAC (47 patients) database. The correlations between lymph node metastasis and molecular features at proteome levels were investigated. Potential immunotherapy targets were identified and prioritized using an *in silico* screening algorithm for cytomembrane proteome.

**Results:**

The *in silico* screening algorithm for cytomembrane proteome led to the recognition of EpCAM and MGST1 as potential targets. We demonstrated that EpCAM and MGST1 were abundantly expressed in LSCC, particularly in cases with lymph node metastasis. Functional siRNA knockdown confirmed their critical roles in driving *in vitro* proliferation, invasion, and migration. Furthermore, their knockdown hindered the Wnt/β-catenin and PI3K signaling pathways.

**Conclusion:**

Integrated cytomembrane proteomics in metastatic LSCC unveils EpCAM/MGST1 as actionable immunotherapeutic targets, with silencing attenuating oncogenic proliferation, invasion, and Wnt/β-catenin-PI3K crosstalk, offering novel therapeutic avenues.

## Introduction

Laryngeal carcinoma accounts for approximately 20% of all head and neck malignancies (Karatzanis et al., 2014) and ranks as the second most common malignancy in the upper respiratory tract, following lung cancer (Megwalu and Sikora, 2014). It is estimated that laryngeal squamous cell carcinoma (LSCC) comprises approximately 85%–95% of laryngeal carcinoma cases, with smoking and alcohol consumption remain the primary risk factors (Steuer et al., 2017). The overall 5-year survival rate for LSCC remains around 50% despite therapeutic advances (Koontongkaew, 2013). Surgery, radiation therapy, and chemotherapy are commonly utilized as the first-line treatment options for LSCC (Hou et al., 2021). However, these conventional treatment strategies often cause significant distress for patients and may not eradicate the probability of recurrence, particularly in cases with severe invasion of surrounding tissues or lymph node metastasis (van den Bosch et al., 2016). Given this, there is a strong need to develop precise and effective therapeutic targets to bolster the power of LSCC treatment.

Although patients diagnosed with early-stage LSCC consistently exhibit favorable treatment outcomes, more than 60 percent of patients diagnosed with advanced LSCC are susceptible to lymph node metastasis. Among these patients, 40% experience recurrence and distant spread (Steuer et al., 2017; Zheng et al., 2017). Ipsilateral single-node metastasis is associated with a 50% decrease in survival, while contralateral or bilateral metastasis further contributes to an additional 50% decrease (Ali et al., 2015; Enepekides et al., 1999). Therefore, the prevention and inhibition of lymph node metastasis should be an essential strategy for controlling tumor progression. Lymph node metastasis is a multi-step process that involves invasion, lymphangiogenesis, lymphatic spread, transportation of cancer cells into the lymph nodes, and their settlement and expansion (Karaman and Detmar 2014). The premise of lymph node metastasis revolves around lymphangiogenesis, which entails the survival, proliferation, and migration of lymphatic endothelial cells (LECs) (Petrova and and Gou, 2020). Mechanically, it has been reported that intratumoral expression of the vascular endothelial growth factor C (VEGF-C) is highly associated with lymph node metastasis (Dieterich et al., 2022). This can be attributed to the significant role of the VEGF-C-VEGFR3 axis in lymphangiogenesis (Oliver et al., 2020). Concordantly, studies have shown that overexpression of COX-2 could stimulate VEGF-C, thereby increasing the risk of lymph node metastasis in head and neck squamous cell carcinoma (HNSC) (Kyzas et al., 2005).

Membrane proteins (MPs) are essential for many cellular processes (Gong et al., 2019), such as bioactive molecule transport, immune system molecular recognition, cell-cell signaling, ion transport, and energy transfer (Gromiha and Ou, 2014). The accessibility of these proteins makes them tractable for targeting in cancer therapy, but identifying suitable targets remains challenging (Gong et al., 2019). Studies and trials on MPs have provided numerous new drugs and potential methods for treating many illnesses, including cardiovascular diseases, psychiatric disorders, certain types of cancer, HIV/AIDS, and more (Sloop et al., 2018). Furthermore, it has been suggested that epithelial MP family members are involved in cell growth and motility, which are essential for cancer progression and metastasis (Ahmat et al., 2019). Monoclonal antibodies targeting MPs not only inhibit cell activity by blocking the function of target proteins, but also potentially offering the transport of cytotoxic components to tumor tissues (Roslan et al., 2022). Although several MPs have been reported in laryngeal cancer (Klobučar et al., 2016), their regulatory mechanisms and potential as therapeutic targets are largely uncovered.

Proteogenomics involves merging next-generation DNA and RNA sequencing with mass spectrometry-based proteomics to comprehensively measure proteins and post-translational modifications for tumor profiling (Ruggles et al., 2017). Currently, various proteomics tumor profiles have been published, including breast cancer (Krug et al., 2020), lung cancer (Chen et al., 2020), hepatocellular carcinoma (Gao et al., 2019), colorectal cancer (Vasaikar et al., 2019), and head and neck squamous cell carcinoma (Huang et al., 2021), among others. Here we conducted a personalized secondary analysis of proteogenomics data from the HNSC-LSCC cohort, aiming to provide a comprehensive perspective on the molecular mechanisms underlying lymph node metastasis in LSCC. The mechanism of these proteins in regulating lymph node metastasis was investigated by performing functional experiments *in vitro*. We present our data to highlight novel targets for LSCC therapy and as an example of an experimental approach that can be utilized to identify personalized immunotherapy targets.

## Materials and methods

### Data collection

Data from laryngeal cancer patients was obtained from The Cancer Genome Atlas (TCGA) and Clinical Proteomic Tumor Analysis Consortium (CPTAC) (Huang et al., 2021). The processed data were accessed via LinkedOmics: http://www.linkedomics.org, including transcriptome, proteome, and corresponding clinical features of all patients from these two datasets. Samples lacking significant clinicopathological or survival information were excluded from further analysis.

### Prognostic risk signature identification

Single-variable Cox proportional hazards regression analysis was conducted to identify features that were significantly associated with overall survival (OS) or progression-free survival (PFS) in LSCC cohorts. The results were visualized using a forest plot created with the R package “ggplot2”. The Kaplan-Meier plotter utilizing the R package “survival” was employed to compare the OS or PFS times between specific groups.

### Tumor mutation analysis

The somatic mutation files were obtained from the TCGA and CPTAC databases. We utilized the “Maftool” R package to display a waterfall plot visualizing the top 20 genes with the highest tumor mutation frequency (TMF) in LSCC patients from TCGA and CPTAC (Mayakonda et al., 2018). Different colors were used to annotate specific mutation types. To evaluate the correlation between lymphatic metastasis and mutations, a chi-square test was conducted, and a *P*-value <0.05 was considered statistically significant.

### Differential expression analysis and functional enrichment

The “Limma” package of R software was applied to evaluate the differential expression proteins (DEPs) and differential expression genes (DEGs) between lymphatic metastasis and non-lymphatic metastasis LSCC (Ritchie et al., 2015). The DEPs were defined as proteins with P-value <0.05 and log2 (Fold Change) > 0.585. The DEGs were defined as genes with P-value <0.05 and log2 (Fold Change) > 1. Functional enrichment analysis, including gene ontology (GO) and the Kyoto Encyclopedia of Genes and Genomes (KEGG), was performed using the DAVID bioinformatics resources (Sherman et al., 2022). The Benjamin–Hochberg adjusted *P* < 0.05 was regarded as statistically significant.

### Subcellular annotation and epitope prediction

The DEPs and DEG encoded proteins were annotated based on the cellular component of the Gene Ontology. The frequency of proteins found in different locations, such as the cytosol, membrane, nucleus, cytoskeleton, mitochondria, Golgi apparatus, and lysosome, is illustrated in the bubble diagram. For epitope prediction, the PDB file of the protein intended for analysis was downloaded from the AlphaFold Protein Structure Database. Afterwards, the files were used as input for Ellipro (http://tools.iedb.org/ellipro/) to estimate the frequency of discontinuous epitopes for each structure (Ponomarenko et al., 2008).

### Prioritization of immunotherapy targets

The plasma MPs that were overexpressed in lymphatic metastasis LSCC were analyzed using the algorithm developed by Anderson et al. for prioritizing immunotherapy targets (Anderson et al., 2022). However, some slight modifications were made to adapt the algorithm to our specific study. The following were determined: fold-change of expression in tumor compared to normal; significance of the difference in expression between the lymphatic metastasis group and the non-lymphatic metastasis group; and off-tumor expression according to Human Proteome Map (Kim et al., 2014). To ensure that each of the three features had a range of 0–1, they were individually scaled based on their maximum value. For each protein, the scaled values were converted into a three-dimensional vector in R3. The final score was then calculated based on the magnitude of this vector, which was capped at a maximum value of approximately 1.732.

### Gene set enrichment analysis (GSEA) and PPI network construction

For gene set enrichment analysis (GSEA), the patients were divided into high- and low-expression groups according to the median protein abundance. GSEA was performed to identify the primarily enriched pathways using the “clusterProfiler” package of R software (Wu et al., 2021). Pathways with the nominal P < 0.01 were considered statistically significant. Protein-protein interaction (PPI) networks were constructed using the STRING database (v.11.5) (Szklarczyk et al., 2019). The minimum required interaction score was set to a threshold of 0.400.

### Cell culture and transfection

LSCC cell lines (TU212 and TU686) were purchased from the Cell Center of Life Science of Chinese Academy of Science (Shanghai, China). TU212 and TU686 cells were maintained in RPMI-1640 medium (Gibco, Carlsbad, CA), supplemented with 10% fetal bovine serum (FBS, Gibco, United States) and 1% penicillin/streptomycin. All cell lines were incubated and cultured at 37°C and 5% CO_2_. EpCAM siRNA (siEpCAM), MGST1 siRNA (siMGST1) and their respective negative controls (siNC) were purchased from Sangon (Shanghai, China). For transfection, cells were seeded onto plates and transfected with specific siRNA using Lipofectamine 3,000 reagent (Invitrogen, Carlsbad, CA, United States) following the manufacturer’s instructions.

### Cell proliferation assay

LSCC cells were seeded in 150 µL of medium in 96-well plates and incubated at 37°C and 5% CO_2_ for 24 h. Next, cells were incubated in 3-[4, 5-dime-thylthiazol-2-yl]-2, 5-diphenyl tetrazolium bromide (MTT) solution (5 mg/mL) for 4 h in a humidified incubator. After dimethyl sulfoxide (DMSO) was added to each well, absorbance was measured with a microplate reader at 570 nm. The experiment was performed at least three times (Bio-Rad, Hercules, CA).

### Western blot analysis

Tu212 and Tu686 cells were collected and washed with PBS, lysed in RIPA buffer containing PMSF (phenylmethylsulfonyl fluoride) and protease and phosphatase inhibitors on ice for 30 min. Proteins were separated by SDS-PAGE and transferred to a polyvinylidene fluoride (PVDF) membrane. Next, the membrane was blocked with a 5% nonfat milk, then incubated with primary antibodies at 4°C overnight. The primary antibodies were used: GAPDH (AC001), EpCAM (A19301), and MGST1 (A0880) from ABclonal. After washing with PBS + Tween 20, cells were incubated with the appropriate secondary antibodies for 2 h at room temperature. Protein bands were detected with ECL (enhanced chemiluminescence) reagents and the ChemiDoc MP imaging system (Bio-Rad). ImageJ software was utilized to perform protein quantification.

### Transwell assay

The migration and invasion of TU212 and TU686 cells were evaluated by transwell assay. For the migration assay, cells were cultured in serum-free RPMI 1640 media in the upper chamber (8-μm pore size, Corning Inc.; New York, United States) and 600 µL medium containing 20% FBS was placed in the lower chamber. After incubation at 37°C and 5% CO_2_ for 24 h, cells were fixed with a 4% PFA solution and stained with 0.5% crystal violet for 20 min. For the invasion assay, the protocol was similar to the migration assay but for cells were grown in Matrigel-coated chambers. The number of migrated and invasive cells was photographed and counted across 5 random fields under an inverted microscope.

### Statistical analysis

Continuous variables were compared by Student’s t-test, while the categorical variables were compared using the chi-square test. The Kaplan-Meier method was used to generate OS and PFS curves, and the log-rank test was performed. Statistical analyses were performed using R version 4.3.2. A *P*-value <0.05 (two sides) was considered statistically significant.

## Results

### Lymphatic metastasis predicted LSCC prognosis

**FIGURE 1:**
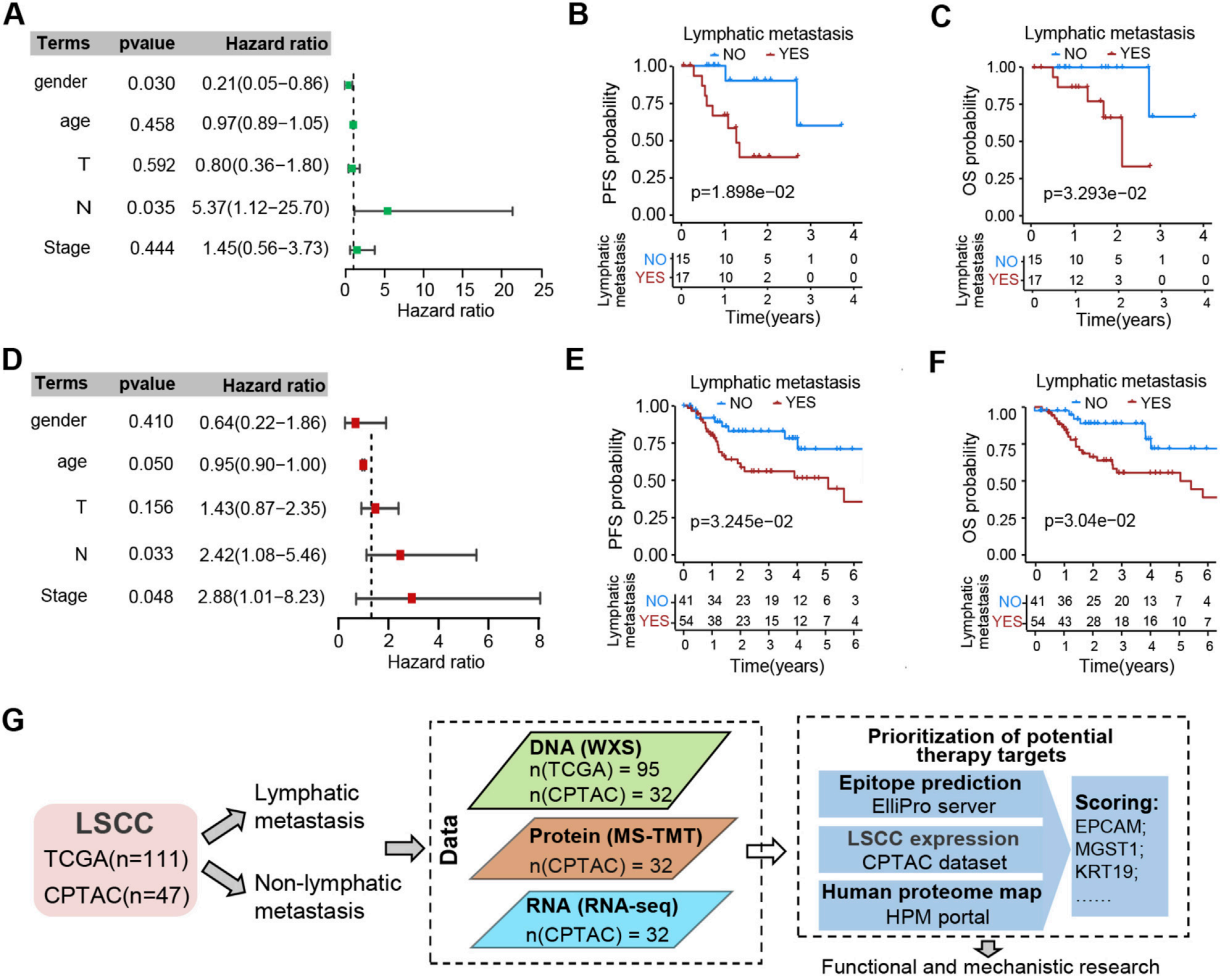
Associations between lymphatic metastasis and outcomes. **(A)** Forest map displays the hazard ratio (HR) and p-value resulting from univariable Cox HR regression analysis of the five independent prognostic factors on PFS. The data pertains to LSCC cases obtained from the CPTAC-HNSC datasets. The significance level for the p-value was set at 0.05. **(B,C)** Kaplan-Meier curves demonstrate PFS **(B)** and OS **(C)** in CPTAC-HNSC-LSCC cohort with or without lymphatic metastasis in their tumor sample. **(D)** Forest map was executed similarly to **(A)**. The data pertains to LSCC cases obtained from the TCGA-HNSC datasets. The significance level for the p-value was set at 0.05. **(E,F)** Kaplan-Meier curves demonstrate PFS **(E)** and OS **(F)** in TCGA larynx cases with or without lymphatic metastasis in their tumor sample. **(G)** Flowchart illustrates the workflow for the integrative analysis of this study, showcasing the samples and omics data used, alongside the schematic diagram for assessing the prioritization of potential therapy targets. Detailed data Supplementary File 2.

Lymph node metastasis is widely recognized as a crucial factor contributing to a poor prognosis among tumor patients. The baseline characteristics of LSCC patients from the TCGA database and CPTAC are shown in Supplementary Table S1. The univariate independent prognostic analysis was conducted on CPTAC-LSCC and TCGA-LSCC cohorts, which showcased a significant correlation between lymph node metastasis and prognosis in LSCC (Figures 1A,D). The patients were then divided into two groups based on the presence or absence of lymph node metastasis. The group without lymph node metastasis demonstrated a longer PFS in comparison to the group with lymph node metastasis, with P-values of 0.01898 for CPTAC cohort and 0.03245 for TCGA cohort. (Figures 1B,E). Consistently, the OS of the group without lymph node metastasis significantly surpassed that of the group with lymph node metastasis, with P-values of 0.033 for CPTAC cohort and 0.030 for TCGA cohort (Figures 1C,F). In line with the current consensus, both TCGA-LSCC and CPTAC-LSCC cohorts displayed the poor prognosis of patients with lymph node metastasis. This suggests that these cohorts are appropriate for investigating the mechanisms of lymph node metastasis and identifying novel therapeutic targets for LSCC. In light of this, gene expression data from 111 LSCC cases was obtained from the TCGA database, while protein expression data from 47 LSCC cases was collected from the CPTAC database. As shown in Figure 1G, a proteogenomic analysis workflow was conducted to examine gene mutations and protein expressions associated with lymph node metastasis. The cell MPs associated with lymph node metastasis were identified and assessed for their potential as therapeutic targets. Additionally, *in vitro* functional experiments were carried out to investigated the underlying mechanisms through which these proteins may regulate lymph node metastasis.

### Landscape of gene mutations and DEPs in lymph node metastatic LSCC

It has been reported that certain gene mutations are correlated with the risk of lymph node metastasis in some types of tumors. To investigate the mutation events related to lymph node metastasis in laryngeal cancer, we acquired whole-exome sequencing data of the TCGA-LSCC and CPTAC-LSCC cohorts. Then the correlation between the top 20 genes with the highest mutation frequencies and lymph node metastasis were analyzed. The results revealed that certain genes had significantly higher mutation frequencies in the non-lymphatic metastasis group compared to the lymphatic metastasis group. These genes include *LRP1B* (*P* = 0.000360), *NSD1* (*P* = 0.033), *USH2A* (*P* = 0.007), *FAT1* (*P* = 0.026), *PAPPA2* (*P* = 0.046), and *RYR2* (*P* = 0.048) (Supplementary Figures 1A,B). Among these, the *LRP1B* and *USH2A*, which are common mutant genes across various cancer types (Brown et al., 2021; López et al., 2023; Möhrmann et al., 2022; Yang et al., 2023), were found to be highly expressed in the lymphatic metastasis group (Supplementary Figure 1C) (Han et al., 2021). The low expression or mutation of *LRP1B* has been reported to be potentially responsible for a lower risk of lymphatic metastasis, which aligns with our findings in this study (Han et al., 2021).

**FIGURE 2:**
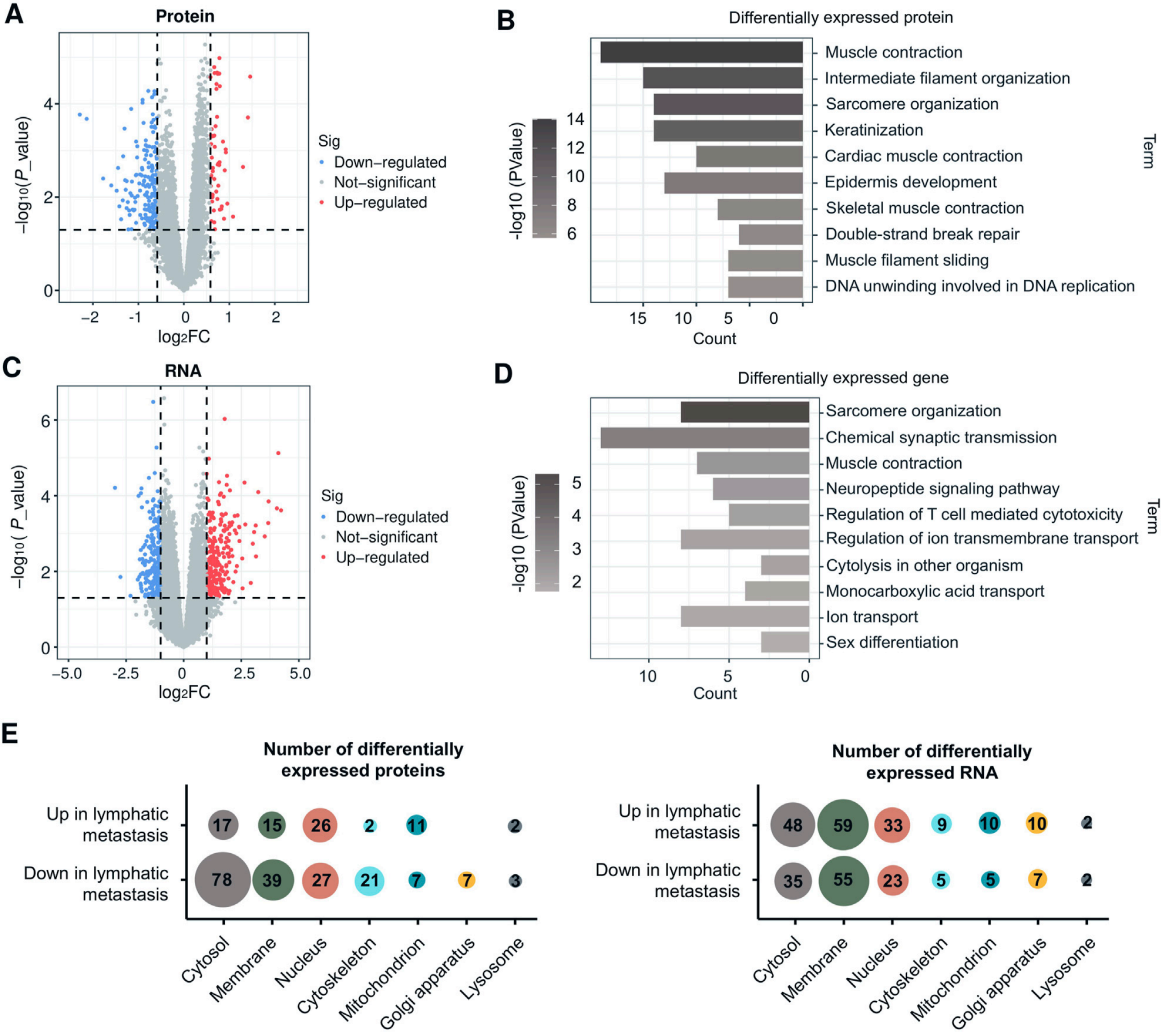
Integrative analysis of the proteome and transcriptome reveals the crucial role of MPs in lymphatic metastasis. **(A)** Volcano plot indicating proteins overexpressed in lymphatic metastasis group or non-lymphatic metastasis (Red and blue colors indicate p < 0.05 (sig) whereas red and blue further require more than 1.5-fold change, other genes are colored in gray). The LSCC proteome data is obtained from the CPTAC-HNSC datasets. **(B)** GO enrichment analysis (Biological process) of DEPs. **(C)** Volcano plot indicating mRNA overexpressed in lymphatic metastasis group or non-lymphatic metastasis group (Red and blue colors indicate p < 0.05 (sig) whereas red and blue further require more than 2-fold change, other genes are colored in gray). The LSCC transcriptome data is obtained from the CPTAC-HNSC datasets. **(D)** GO enrichment analysis (Biological process) of DEGs. **(E)** Bubble chart showing breakdown of upregulated and downregulated proteins and mRNAs in lymphatic metastasis compared with non-lymphatic metastasis by cell compartment. Detailed data Supplementary File 3.

To gain a comprehensive understanding of the regulatory factors associated with lymph node metastasis in LSCC, we performed an integrative analysis on the proteome and corresponding transcriptome of the CPTAC-LSCC cohort. By conducting differential expression analysis between the non-lymphatic metastasis group and the lymphatic metastasis group, we discovered 228 differentially expressed proteins (DEPs) including 54 upregulated proteins and 174 downregulated proteins (with a fold change >1.5 and p < 0.05) (Figure 2A). Additionally, 535 differentially expressed genes (DEGs), consisting of 277 upregulated genes and 258 downregulated genes (with a mRNA fold change >2 and p < 0.05) were identified (Figure 2C).

Functional enrichment analysis was conducted using the DAVID service to generate representative biological processes involved in lymph node metastasis. As depicted in Figures 2B,D, the DEPs and DEGs were primarily enriched among terms such as “muscle contraction,” “intermediate filament organization,” “sarcomere organization,” “keratinization,” and “epidermis development.” These processes are closely associated with cell growth and motility. The DEPs and DEGs were then classified based on their subcellular localizations, using the Cell component (CC) catalog of the Gene Ontology (GO) database. Majority of DEPs were localized in the cytosol, cell membrane, and nucleus (Figure 2E). Given the accessibility of MPs in the targeted therapy or immunotherapy, our interest has been centered around the highly expressed MPs found in the lymph node metastasis group. These proteins have the potential of serving as therapeutic targets for lymph node metastatic LSCC.

### Identification of novel therapeutic targets based on membrane proteome profiling

**FIGURE 3:**
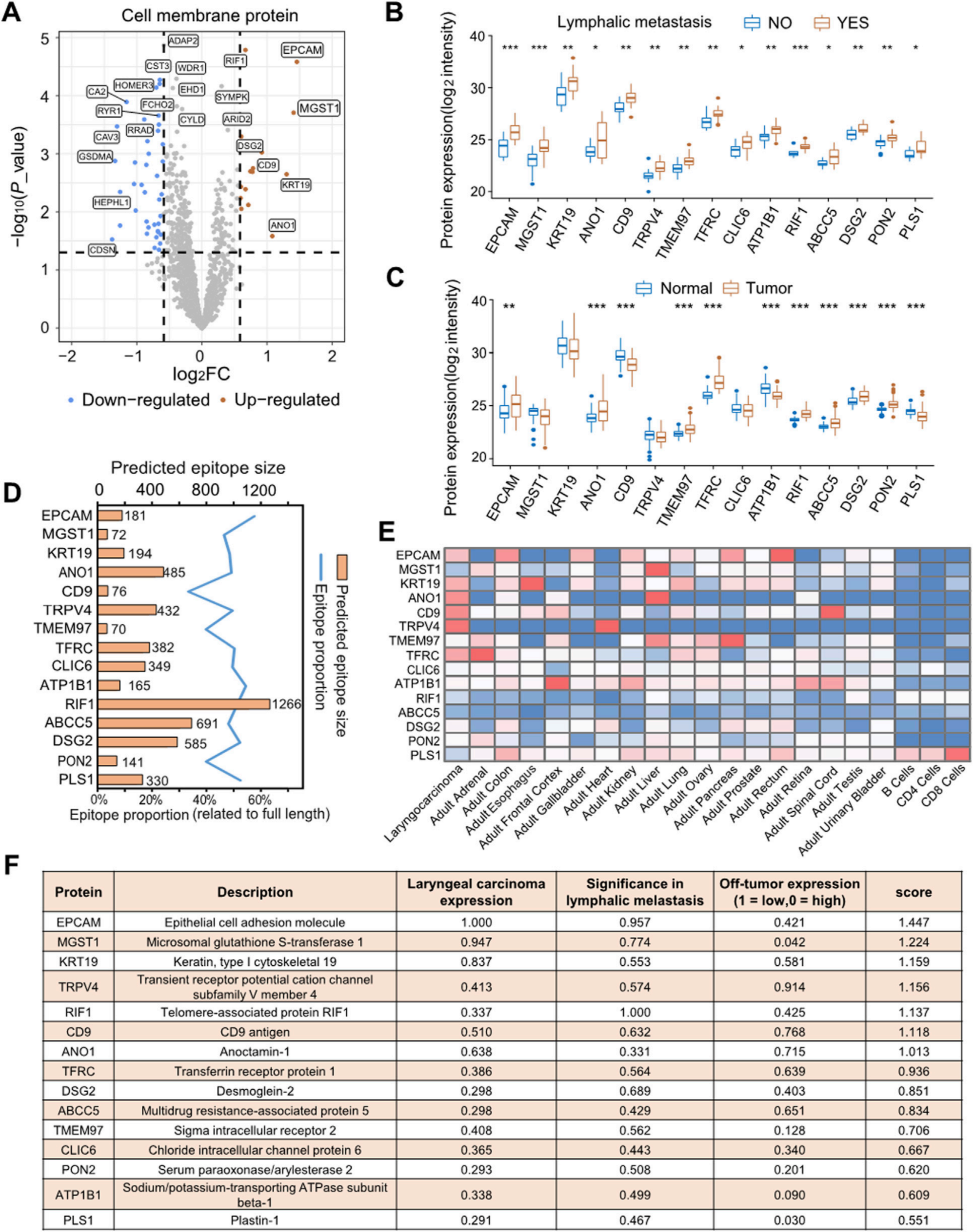
MPs analysis to identify candidate therapeutic targets for laryngeal carcinoma. **(A)** Volcano plot displays differential MP expression between lymphatic metastasis group or non-lymphatic metastasis group. **(B)** Box plots display protein expression values for 15 MPs related to lymphatic metastasis. The expression level was transformed using log2 before conducting the t-test. The symbols “*”, “**”, and “***” signify p < 0.05, p < 0.01, and p < 0.001, respectively. **(C)** Box plots display the expression levels of 15 proteins in both tumor and tumor-adjacent tissues. The expression levels were transformed using log2 before conducting the t-test. The symbols “*”, “**”, and “***” signify p < 0.05, p < 0.01, and p < 0.001, respectively. **(D)** Antigen epitope analysis of 15 highly expressed MPs. **(E)** The expression profiling of 15 MPs in LSCC and other human tissues in Human Proteome Map (HPM) (www.humanproteomemap.org). **(F)** Prioritization of potential therapeutic targets for LSCC with lymphatic metastasis. “Laryngeal carcinoma expression” refers to the fold-change of expression in tumor compared to normal, determined by proteome profiling in CPTAC, standardized to a maximum fold-change of 1. “Significance in lymphatic metastasis” is determined by the p-value resulting from the differential analysis of the lymphatic metastasis group and the non-lymphatic metastasis group. The obtained values are then log transformed and standardized with a maximum value of 1. “Off-tumor expression” refers to expression across multiple tissues in the HPM database, it is taken as 1 if there is no expression in any of the tissues, or if there is expression, it is calculated as the negative log of the maximum tissue expression in arbitrary units/10. The “score” represents the magnitude of the vector formed by “Laryngeal carcinoma expression”, “Significance in lymphatic metastasis”, and “Off-tumor expression” and is limited to a maximum value of 3^0.5 (∼1.732). Detailed data Supplementary File 4.

We have previously discovered that some of the DEPs related to lymph node metastasis are located on the cell membrane (Figure 2E). Differential analysis was conducted on all quantified cell MPs within the proteome data, resulting in the identification of 15 cell MPs that were overexpressed in the tumors with lymph node metastasis and 39 downregulated MPs (Figure 3A). The segment of the MP that is exposed on the cell surface is a highly promising drug target, as approximately 60% of drug targets are MPs (Overington et al., 2006). Figure 3B displays the expression level of 15 metastasis-specific MPs, the majority of which exhibited overexpression in tumor tissues in comparison to normal tissues (Figure 3C). To verify their potency as therapeutic targets, we first performed antibody epitope prediction using three-dimensional protein structure models generated by the Alphafold database, utilizing the ElliPro server. The results demonstrated that these proteins possess antigenic epitopes of at least 70 amino acids or more, making them susceptible to targeting by monoclonal antibodies (Figure 3D). Subsequently, a prioritization of these 15 MPs as immunotherapy targets was conducted, taking into account their distribution across various organs, abundance in laryngeal carcinoma, and correlation with lymph node metastasis (Figures 3E,F). Epithelial cell adhesion molecule (EpCAM) and Microsomal glutathione S-transferase 1 (MGST1) are the top-scoring proteins, exhibiting significantly higher scores than others (Figure 3F).

### EpCAM and MGST1 are prognostic markers for laryngeal carcinoma

**FIGURE 4:**
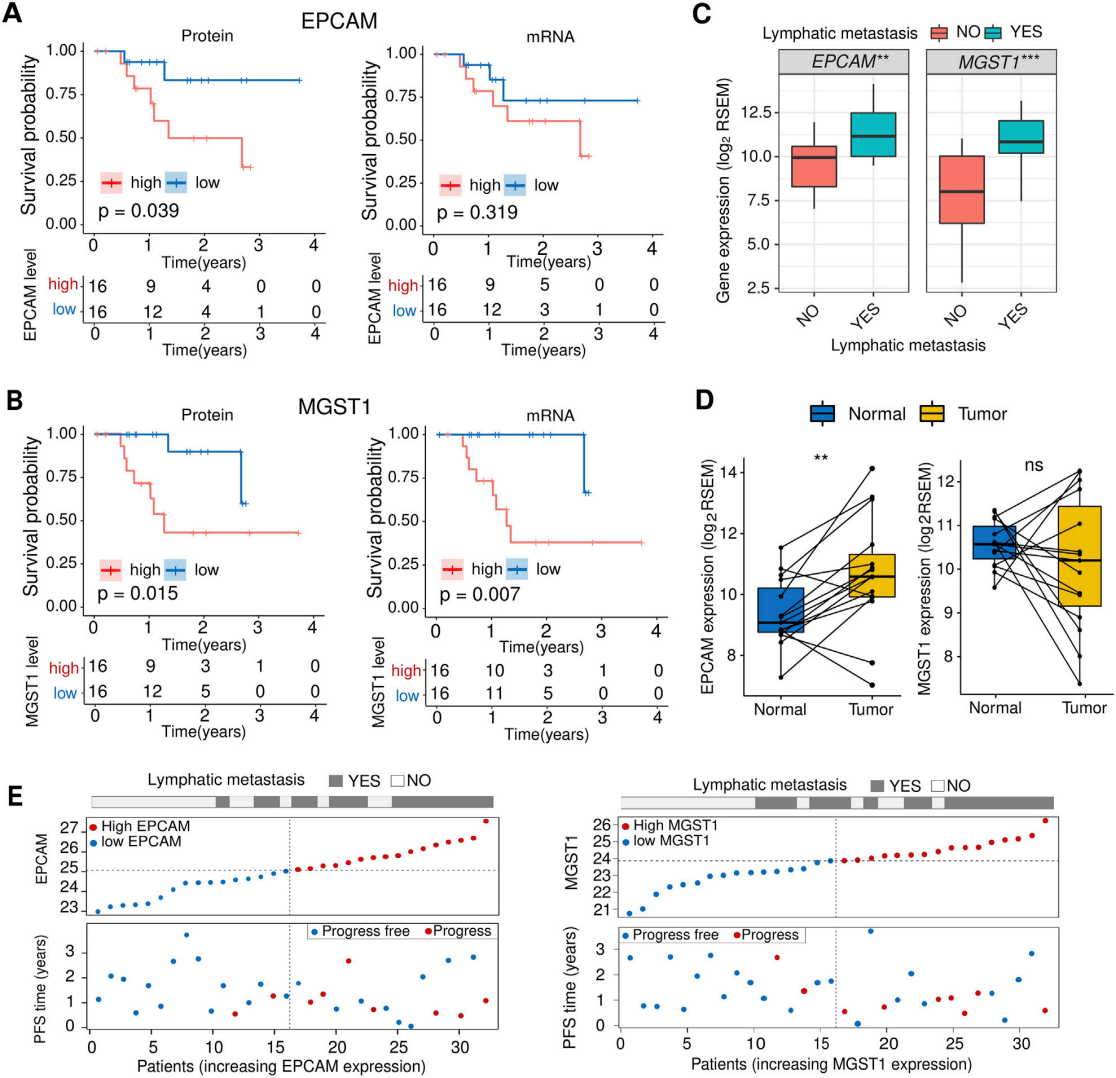
EpCAM and MGST are associated with prognosis. **(A)** Kaplan-Meier plot comparing OS for patients stratified by the median EpCAM protein (left) or mRNA (right) level. The p values were derived from log rank tests. **(B)** Kaplan-Meier plot comparing OS for patients stratified by the median MGST1 protein (left) or mRNA (right) level. The p values were derived from log rank tests. **(C)** Comparison of mRNA expression levels of EpCAM and MGST1 between the lymphatic metastasis group and the non-lymphatic metastasis group. **(D)** Comparison of mRNA expression levels of EpCAM and MGST1 in tumor tissues and adjacent normal tissues. **(E)** The risk curve (top) of each sample is reordered based on the protein expression levels of EpCAM (left) or MGST1 (right). The scatter plots of the sample’s PFS overview are displayed in the bottom panel, with the blue and red dots representing progression-free and progression, respectively.

Since EpCAM and MGST1 were found to be overexpressed in lymphatic metastatic LSCC, we investigated the prognostic relevance of these proteins. Our findings showed that patients with overexpression of EpCAM or MGST1 had a worse prognosis (Figures 4A,B). EpCAM and MGST1 showed significant overexpression at both the protein and mRNA levels in lymphatic metastatic LSCC (Figure 4C). It is also noteworthy that expression of EpCAM was higher in cancer tissue compared to adjacent tissue. On the other hand, MGST1 was only upregulated in lymphatic metastatic LSCC, with no significant difference between cancer and adjacent tissue (Figure 4D). Furthermore, the risk curve illustrates a positive correlation between EpCAM and MGST1 with disease progression, which aligns with the findings of the survival curve (Figure 4E).

### EpCAM and MGST1 are required for cell proliferation, migration, and invasion

**FIGURE 5:**
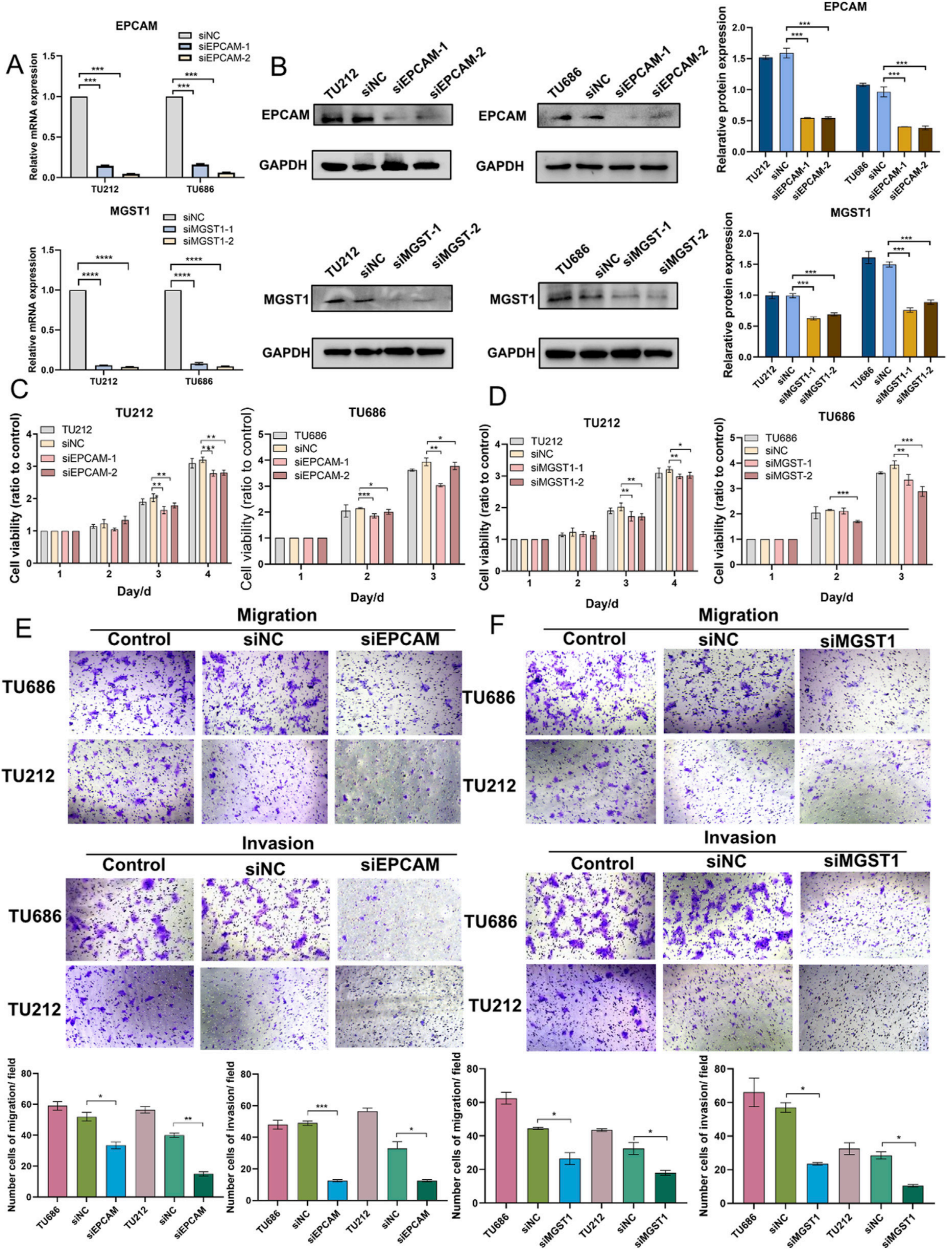
Effects of EpCAM and MGST1 in Tu686 and TU212 cells. **(A)** The mRNA expression of EpCAM and MGST1 were examined in TU686 and TU212 cells after transfection with siRNA, as well as in control cells. **(B)** The protein expression of EpCAM and MGST1 in TU686 and TU212 cells **(C,D)** MTT assay was performed to examine proliferation of TU686 and TU212 cells after knockdown of EpCAM **(C)** and MGST1 **(D)**. **(E,F)** Transwell migration and invasion assay were conducted in TU686 and TU212 cells transfected with EpCAM siRNA **(E)** and MGST1 siRNA **(F)**. Data are presented as the mean ± SD.

To explore the role of EpCAM and MGST1 in LSCC cells, TU212 and TU686 cells were separately transfected with siRNA-targeting EpCAM, siRNA-targeting MGST1, or non-specific RNAi control (Figure 5A). Western blot showed more than 50% knockdown efficiency in TU212 and TU686 cells (Figure 5B) for both EpCAM and MGST1. MTT assays showed that silencing EpCAM or MGST1 significantly reduced the proliferation of TU686 and TU212 cells (Figures 5C,D). The transwell assay demonstrated that the reduction of EpCAM leads to a significant decrease in the migration and invasion of LSCC cells (Figure 5E), and similar effects were observed with MGST1 knock-down (Figure 5F). These results collectively provided evidence that the deficiency of EpCAM or MGST1 suppresses the proliferation, migration, and invasion of LSCC cells. These proteins might therefore represent promising therapeutic opportunities in attempts to improve the poor prognoses of lymphatic metastatic LSCC.

### Potential regulatory mechanisms of EpCAM and MGST1 in CPTAC laryngeal cancer

**FIGURE 6:**
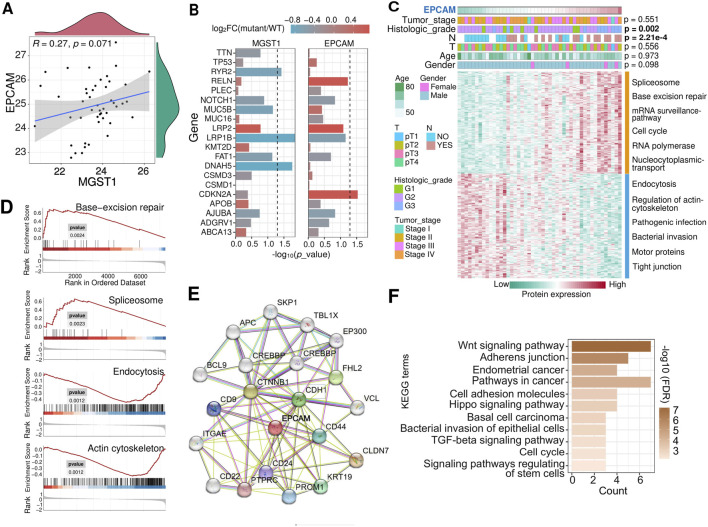
Analysis of potential regulatory mechanisms of EpCAM in laryngeal cancer. **(A)** Correlation between the abundance of the MGST1 and EpCAM proteins. **(B)** Correlation of EpCAM and MGST1 protein expression with genetic mutations in tumors. The horizontal line denotes an p value cutoff of 0.05. **(C)** Heatmap showed proteins that were positively or negatively correlated with EpCAM in CPTCA-LSCC patients with varying clinicopathological characteristics. The enriched KEGG pathway terms of the correlation proteins were demonstrated. **(D)** GSEA of proteins across high and low EpCAM expression groups shows enriched pathways, corresponding to KEGG enrichment in **(C)**. **(E)** PPI showed the proteins that interact with EpCAM. **(F)** KEGG pathway enrichment analysis of EpCAM interacting proteins.

EpCAM is regarded as a carcinoma cell-surface marker involved in cell adhesion, proliferation, migration, stemness, and epithelial-to-mesenchymal transition. MGST1 is a membrane-bound glutathione transferase with the ability to detoxify reactive intermediates. Our findings demonstrated that overexpression of EpCAM and MGST1 are closely correlated with lymphatic metastasis and play crucial role in driving cell growth, migration, and invasion. However, there was no significant correlation between the expression of these two proteins (Figure 6A), implying distinct mechanisms by which EpCAM and MGST1 are likely involved in lymphatic metastasis. Here we aimed to examine the regulatory mechanisms behind the disordered expression of these proteins in LSCC.

Patients with high EpCAM featured more CDKN2A mutations and tumors of higher histologic grade (*p* = 0.002) (Fisher’s exact test) (Figures 6B,C). Tumors with high EpCAM showed specific elevation in pathways involving spliceosome and mismatch repair, and downregulation in pathways related to endocytosis and regulation of the cytoskeleton (Figures 6C,D). It can be inferred from Figure 4 that the overexpression of EpCAM, along with its facilitation of lymph node metastasis, could be associated with a protein-level regulatory mechanism, such as PPI. Given this, PPI analyses were carried out by STRING server (Figure 6E), the EpCAM-interacting proteins were mainly enriched in cancer-related biological processes, including the Wnt signaling pathway, adhesion junctions, and the Hippo signaling pathway (Figure 6F). Patients with low MGST1 were characterized by harboring more RYR2, LRP1B, or DNAH5 mutations (Figure 6B). Tumors with high MGST1 showed specific upregulation of pathways relevant to ribosome assembly and oxidative phosphorylation and downregulation of pathways involving extracellular matrix, proteoglycans of binding, protein digestion and absorption (Supplementary Figures S2A,B). The top three proteins showing the highest correlation with MGST1 were SUPT16H, NOP2, and APEX1. Both APEX1 and NOP2 are recognized regulators of tumor metastasis, suggesting a potential mechanism wherein the lymphatic metastasis associated with high MGST1 could be attributed to the overexpression of either APEX1 or NOP2 (Supplementary Figures S2C–E). Taken together, these *in silico* analyses may provide guidance for further research on the regulatory mechanisms of EpCAM and MGST1 in cancers.

### Wnt/β-catenin and PI3K signaling pathways are responsible for EpCAM and MGST1-mediated lymphatic metastasis of LSCC

**FIGURE 7:**
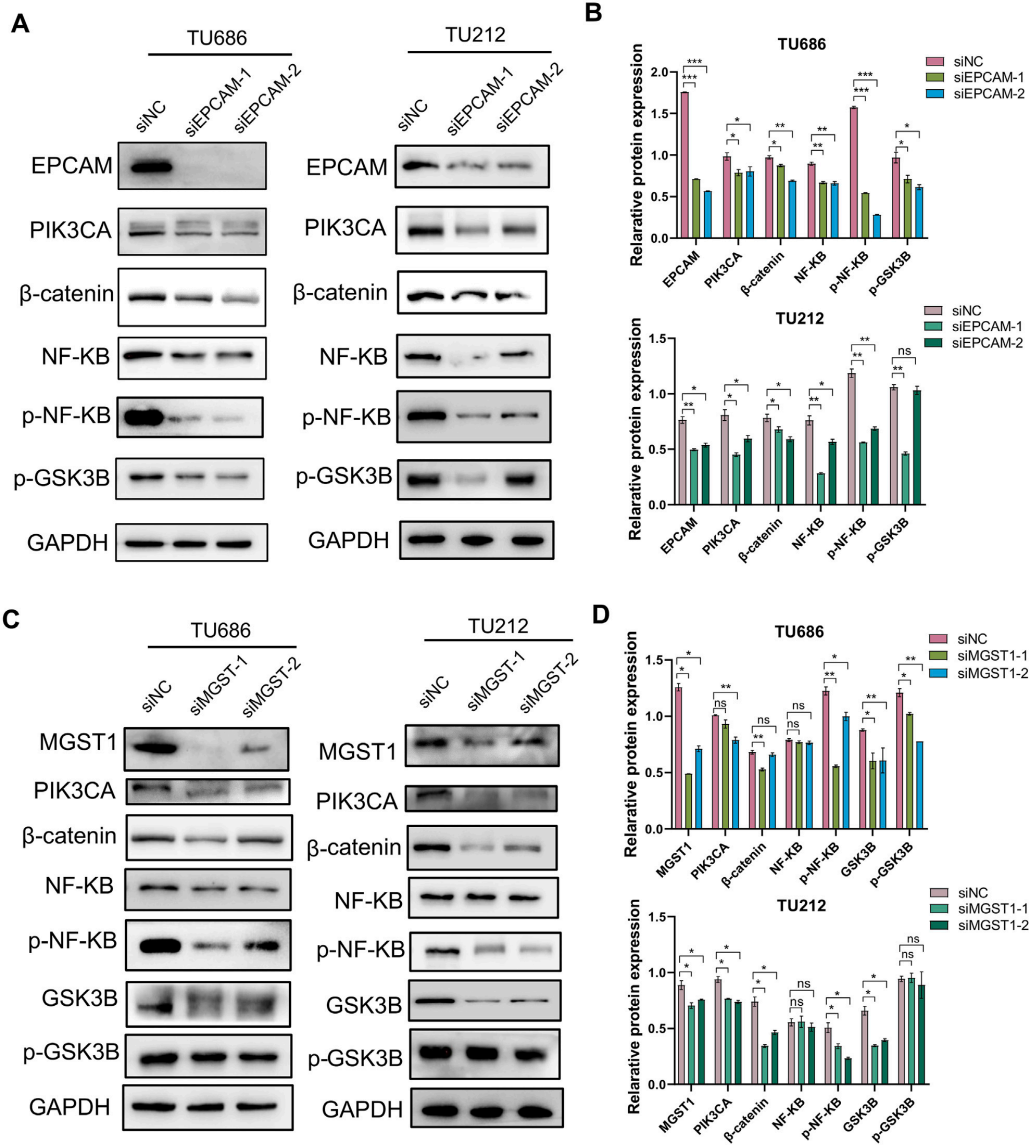
The effects of EpCAM or MGST1 silencing on signaling pathways. Expression levels of PIK3CA, β-catenin, NF-KB and GSK3β in EpCAM **(A,B)** or MGST1 **(C,D)** knockdown cells were measured via Western blot assay.

It has been reported that EpCAM and MGST1 participate in cell signaling, including phosphaditylinositol-3 kinase and wnt/β-catenin pathway (Dai and Lu, 2022; Li, et al., 2023c; Munz et al., 2009; Ni et al., 2013). This is significant due to the support of these pathways in tumor cell proliferation, survival, and anti-apoptotic responses. To investigate the mechanism by which EpCAM or MGST1 regulates the progression of LSCC, we examined the effects of EpCAM and MGST1 knockdown on the wnt/β-catenin and PI3K signaling pathways in TU686 and TU212 cells. As anticipated, silencing EpCAM resulted in downregulation of PIK3CA, NFKB, and phosphorylated NFKB, as well as the inhibition of β-catenin and downstream phosphorylated GSK3β (Figures 7A,B). Of note, knockdown of MGST1 resulted in a similar signaling interference (Figures 7C,D). These findings support the mechanism whereby the two biomarkers, enriched in lymphatic metastatic LSCC, are consistently associated with the Wnt/β-catenin or PI3K signaling pathways, thus leading to tumor-promoting effects.

## Discussion

Lymph node metastasis is an important prognostic indicator of solid tumors (Baek, 2022). Some evidence indicates that lymph nodes actively contribute to the development of distant metastases in vital organs, ultimately resulting in fatality (Kawada and Taketo, 2011). However, our understanding of the molecular mechanisms behind lymph node metastasis in laryngeal cancer is limited.

In this study, we performed a comprehensive proteomic analysis of 111 LSCC samples from the TCGA cohort and 47 LSCC samples from the CPTAC cohort. The patients were grouped into the lymphatic metastasis group and no lymphatic metastasis group. In our analysis of the relationship between lymph node metastasis and gene mutation, we were surprised to observe that the frequency of mutations in the lymph node metastasis group appeared to be lower. In particular, patients with mutations in the *LRP1B* gene had a lower probability of lymph node metastasis (Supplementary File 1). This indicates that intact LRP1B may be essential for the process of lymph node metastasis in LSCC. Supporting this hypothesis, the expression level of LRP1B was significantly higher in the lymphatic metastasis group compared to the group without lymphatic metastasis, and a similar trend has also been observed in esophagogastric junction adenocarcinoma (Han et al., 2021). It is crucial to authenticate these findings in larger prospective studies, and additional experimental investigations are necessary to elucidate the precise mechanism through which LRP1B regulates lymph node metastasis.

A significant proportion of the proteins associated with lymph node metastasis identified in our analysis were found on the cell membrane. MPs can be targeted by drugs as primary or secondary targets, and play a crucial role in determining pharmacological actions due to their specific biological location and characteristics (Hauser et al., 2018). A wealth of research suggests that MPs are vital drug targets for cancer (Ahmat Amin et al., 2019), inflammatory diseases (Wei et al., 2023), schizophrenia (El Fadili et al., 2022) and asthma (Dantzer and Wood, 2019). Given the wide application of cytomembrane proteins as targets for anti-tumor drugs, the 15 MPs overexpressed in lymph node metastasis LSCC in this study were subjected to a ranking algorithm that enabled the discovery of novel immunotherapy targets (Figure 3). The validity of this ranking approach is supported by its identification of EpCAM among the top hits, an established immunotherapy target. EpCAM is now recognized as one of the most studied tumor-associated antigens and has developed into a well-established marker for epithelial cells utilized in pathological examinations of various cancers (Baeuerle and Gires, 2007; de Gouw et al., 2020; Romeu et al., 2013). In our experiments, the role of EpCAM in laryngeal cancer cell proliferation, cell invasion and migration were also confirmed. This implies that EpCAM has the potential to be a target for immunotherapy of lymphatic metastatic LSCC. The protein expression of PIK3CA, p-NF-κB, β-catenin, and p-GSK3β was downregulated after silencing EpCAM (Figure 7A). Furthermore, EpCAM interaction proteins were found to be enriched in the Wnt-β-catenin signal pathway (Figure 6E), supporting the hypothesis that EpCAM overexpression may promote lymph node metastasis of LSCC by activating the Wnt signaling pathway.

EpCAM as a cell-surface tumor-associated antigen, which makes it amenable to antibody-based immunotherapies. Preclinical and clinical studies in other epithelial cancers, such as colorectal (Dutta et al., 2024; Tapia-Galisteo et al., 2022) and ovarian carcinomas (Fu et al., 2021; Li et al., 2023b; Richter et al., 2010), have explored EpCAM-directed approaches, including bispecific T cell engagers (catumaxomab targeting EpCAM x CD3) and chimeric antigen receptor (CAR) T cell therapies. For instance, the anti-EpCAM monoclonal antibody catumaxomab has been approved for malignant ascites in Europe (Syed, 2025), and trials like NCT02915445 are evaluating EpCAM-targeted CAR-T cells in solid tumors (Li et al., 2023a). These advancements suggest that EpCAM could be leveraged in LSCC using existing immunotherapeutic platforms, particularly for tumors with high surface expression.

Based on the ranking algorithm in this study, MGST1 ranked second, with a score slightly lower than EpCAM, making it a novel potential therapeutic target for LSCC. There have been reports of high expression of MGST1 in pancreatic cancer (Kuang et al., 2021), glioma (Yang et al., 2021), and melanoma (Zeng et al., 2020b; Zhang et al., 2023), and correlates with poor prognosis in multiple cancers, underscoring its broad relevance (Morgenstern et al., 2011). Knockdown of MGST1 inhibits lung adenocarcinoma cell proliferation and induces apoptosis via inactivation of the AKT/GSK-3β signaling pathway (Zeng et al., 2020a). The elevation of MGST1 has demonstrated enhanced cell proliferation, migration, invasion of trophoblast by the activation of the PI3K/AKT/mTOR pathway (Dai and Lu, 2022), and activation of the AKT/GSK-3β/β-catenin axis in gastric cancer cells (Li et al., 2023c). Despite this knowledge, the regulatory mechanisms underlying its specific involvement in LSCC, particularly in the context of lymph node metastasis, remained unclear. Toward this end, we investigated MGST1 function in LSCC cells and observed that silencing MGST1 significantly inhibited tumor cell proliferation, migration, and invasion (Figures 5D,F), likely through downstream suppression of PIK3CA, NF-KB, β-catenin and GSK3β (Figure 7C). These findings suggest that the effect of MGST1 on laryngeal cancer cells is partly mediated by the Wnt/GSK-3β/β-catenin signaling pathway. In addition, it was noted that, unlike EpCAM, the differential expression of MGST1 was only observed in cases with lymph node metastasis compared to cases without metastasis. This finding suggests that MGST1 could potentially be a singular therapeutic target specifically for LSCC cases with lymph node metastasis.

MGST1, while less explored as a direct immunotherapy target, represents a candidate for small-molecule inhibition or synergistic approaches due to its role in chemoresistance (Jagust et al., 2020) and oxidative stress regulation (Zhang et al., 2023). Although no MGST1-specific immunotherapies are currently in trials, its interaction with pathways like the NF-κB cascade (often modulated by checkpoint inhibitors) could enable therapeutic synergy (Bravo-Cuellar et al., 2020). Targeting MGST1 alters the redox balance and suppresses metastatic progression in melanoma, thereby improving the efficacy and safety of chemotherapy and immune checkpoint inhibitors (Zhang et al., 2023). Additionally, MGST1 high expression contributed to cisplatin resistance of NSCLC cells by inhibiting ALOX5-induced ferroptosis (Yuan et al., 2025). Collectively, these findings suggest that MGST1 holds significant promise as a therapeutic target. However, further validation, particularly focusing on its pronounced role in metastatic LSCC, could provide a valuable opportunity to advance its clinical translation.

### Limitations of the study

While the integrated proteomic approach identified EpCAM and MGST1 as promising therapeutic targets in metastatic LSCC, this study has limitations that merit consideration: Functional characterization of EpCAM and MGST1 was confined to *in vitro* systems, and *in vivo* validation using appropriate animal models would be valuable to substantiate translational potential. Although integration of TCGA and CPTAC datasets provided a cohort, larger independent cohorts incorporating additional proteomics datasets would strengthen generalizability. Furthermore, while the data suggest modulation of the Wnt/β-catenin-PI3K pathway, elucidation of the precise molecular mechanisms governing EpCAM/MGST1 interactions with these pathways represents an important direction for future investigation.

## Conclusion

Our study characterized the comprehensive proteogenomics of laryngeal carcinoma with lymph node metastasis and analyzed the molecular mechanisms involved. We proposed and demonstrated the value of MPs as potential therapeutic targets for laryngeal cancer. We believe this study offers valuable insights for understanding the progression of LSCC with lymph node metastasis and facilitates advancements in the development of diagnostics and therapeutics for LSCC patients with lymph node metastasis.

## Data Availability

The original contributions presented in the study are included in the article/Supplementary Material, further inquiries can be directed to the corresponding authors.

## Supplementary material

SUPPLEMENTARY TABLE 1 Clinicopathological data of patients in TCGA-HNSC-larynx cohort and CPTAC- HNSC-larynx cohort.

SUPPLEMENTARY FILE 1 Mutational landscape of a TCGA-HNSC-larynx cohort and CPTAC- HNSC-larynx cohort.

SUPPLEMENTARY FILE 2 Analysis of potential regulatory mechanisms of MGST1 in laryngeal cancer.

SUPPLEMENTARY FILE 3 Differential proteomic and transcriptomic analysis of laryngeal carcinoma with and without lymphatic metastasis.

SUPPLEMENTARY FILE 4 Genetic profile of laryngeal carcinoma cases from TCGA and CPTAC database.

SUPPLEMENTARY FILE 5 Cell membrane proteins correlated with lymphatic metastasis.

## Abbreviations

LSCC, Laryngeal Squamous Cell Carcinoma; HNSC, Head and Neck Squamous Cell; MPs, Membrane Proteins; TCGA, The Cancer Genome Atlas; CPTAC, Clinical Proteomic Tumor Analysis Consortium; EpCAM, Epithelial Cell Adhesion Molecule; MGST1, Microsomal Glutathione S-Transferase 1; GO, Gene Ontology; KEGG, Kyoto Encyclopedia of Genes and Genomes; GSEA, Gene Set Enrichment Analysis; PPI, Protein-Protein Interaction; OS, Overall Survival; PFS, Progression-Free Survival; DEPs, Differentially Expressed Proteins; DEGs, Differentially Expressed Genes; MTT, 3-(4,5-Dimethylthiazol-2-yl)-2,5-Diphenyltetrazolium Bromide.
