# Supplementary material for: Integrated cytomembrane proteomics identifies EpCAM/MGST1 as therapeutic targets in metastatic laryngeal carcinoma
Source: Front Genet. 2025 Jul 24;16:1615570. doi: 10.3389/fgene.2025.1615570 (PMC12328148; doi:10.3389/fgene.2025.1615570)
Supplement: Supplementary file 2 [file Table1.docx]

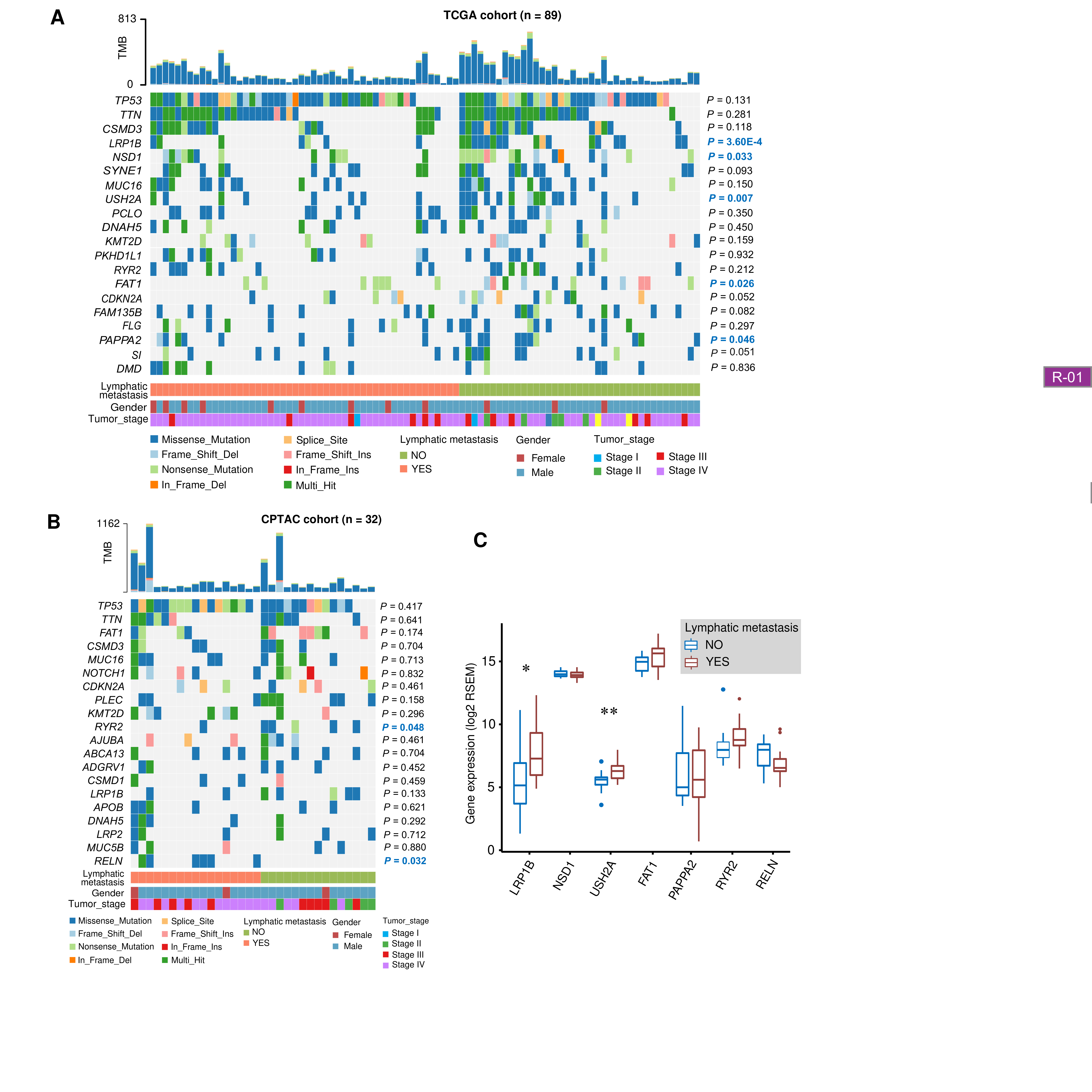


**Figure S1.** Mutational landscape of the TCGA-HNSC-larynx cohort and CPTAC- HNSC-larynx cohort.

(**a, b**) The top 20 gene mutations in the TCGA-HNSC-Larynx cohort (**a**) or CPTAC- HNSC-Larynx cohort (**a**) exhibit various clinicopathological features. The P-values on the right were calculated using the chi-square test to analyze the correlation between gene mutations and lymphatic metastasis. (c) Box plots show the mRNA expression of mutant genes that are correlated with lymphatic metastasis. The genes with a p-value < 0.05 in (a) or (b) are shown. Detailed data Supplementary file 4.
