# Supplementary material for: Integrated cytomembrane proteomics identifies EpCAM/MGST1 as therapeutic targets in metastatic laryngeal carcinoma
Source: Front Genet. 2025 Jul 24;16:1615570. doi: 10.3389/fgene.2025.1615570 (PMC12328148; doi:10.3389/fgene.2025.1615570)
Supplement: Supplementary file 4 [file Table2.docx]

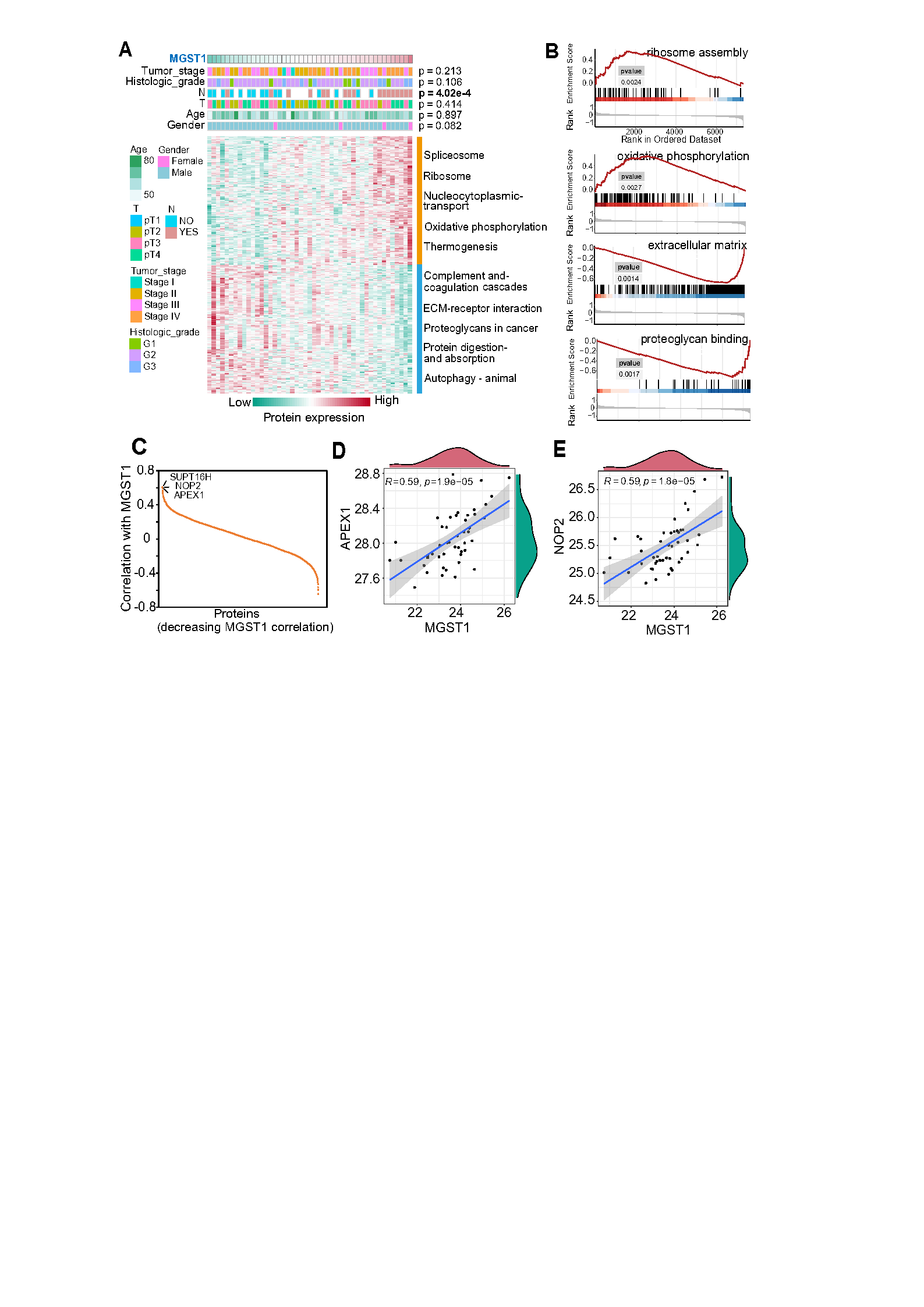


**Figure S2 .** Analysis of potential regulatory mechanisms of MGST1 in laryngeal cancer. (**a**) Heatmap showed proteins that were positively or negatively correlated with MGST1 in CPTCA-LSCC patients with varying clinicopathological characteristics. The enriched KEGG pathway terms of the correlation proteins were demonstrated. (**b**) Gene set enrichment analysis (GSEA) of proteins across high and low MGST1 expression groups shows enriched pathways, corresponding to KEGG enrichment in (**a**). (**c**) Ranking of genes associated with MGST1 expression. The top three proteins with highest correlation coefficient were labeled. (**d, e**) Correlation of protein expression between MGST1 and APEX1(**d**) or NOP2(e).
